# Blastocyst culture in the Era of PGS and FreezeAlls: Is a ‘C’ a failing grade?

**DOI:** 10.1093/hropen/hox017

**Published:** 2017-11-10

**Authors:** Dean E Morbeck

**Affiliations:** 1 Fertility Associates, Auckland, New Zealand; 2 Department of Obstetrics and Gynaecology, University of Auckland, Auckland, New Zealand

Frozen human blastocysts are the currency of modern ART. A strong blastocyst cryopreservation programme is requisite for an effective single embryo transfer policy, to minimize ovarian hyperstimulation syndrome, enhance both the health and number of live born from ART, and apply modern PGS at the blastocyst stage. The number of blastocysts available is a key determinant for a patient’s chance of success with ART, thus blastocyst development rate is an important measure of an IVF clinic’s performance. To this end, the Vienna Consensus on key performance indicators (KPIs) in the ART laboratory is an important step in refining our definition of quality of care (ESHRE SIG/Alpha, 2017).

Blastocyst grading is subjective with significant intra- and inter-user variability, features that place the decision to biopsy or freeze an embryo in a critical role with a direct impact on patient care and KPIs. Since we grade blastocysts with two goals—to assess viability and establish relative quality (rank order)—our decisions have different consequences. For patients with a limited number of embryos, the decision regarding viability carries considerable weight if only grade ‘C’ embryos are available. For patients with an abundance of embryos, the credibility and accuracy of grading may affect time to pregnancy.

Blastocyst grading as it is widely practiced is far from standardized and suffers from limited evidence for effectiveness—both in terms of viability and rank order. This paper discusses the history and current state of blastocyst grading and offers suggestions to address its limitations.

## History of Blastocyst Grading

Blastocyst grading schemes have evolved as clinical practice has shifted from cleavage stage to blastocyst stage transfers and freezing. Blastocysts have been used in ART for more than 30 years (Edwards and Steptoe, 1983) and the first grading scheme was based on degree of expansion (Cohen *et al.*, 1985). Interest in blastocyst quality increased with the introduction of sequential media for culture to the blastocyst stage (Gardner and Lane, 1998). While there are still laboratories that grade blastocysts solely on degree of expansion (Bodri *et al.*, 2015; Braga *et al.*, 2016), the majority of blastocyst grading schemes today are based on the Gardner system (Gardner and Schoolcraft, 1999), which introduced individual grades (A, B, C) for the inner cell mass (ICM) and trophectoderm (TE) in addition to degree of blastocyst expansion (score 1–6). The Gardner grading system introduced complexity with the intent to improve prognostic value. According to the Istanbul Consensus Workshop on embryo assessment (Alpha, 2011a, 2011b), ‘It was anticipated that the scoring system would then be modified and refined once the significance of the scores were understood.’

The Gardner grading system has been modified by several groups to change its complexity. In an effort to address the limited choices for ICM and TE grades, Veeck and Zaninovic (Veeck and Zaninović, 2003) added a fourth grade (D) for degenerative cells. The system was subsequently simplified by changing the alphanumeric grades to numbers to allow a single blastocyst quality score (Rehman *et al.*, 2007). In contrast to these approaches, most embryology societies have simplified the grading system by decreasing the number of expansion grades (from 6 to 4) and naming the ICM/TE grades good, fair and poor (Alpha, 2011a; Racowsky *et al.*, 2011). More recently a single grade of good, fair or poor that is based on Gardner scores has been proposed (Richardson *et al.*, 2015), using grades that are similar to those applied for Top, Intermediate and Low quality blastocysts (Wirleitner *et al.*, 2016).

Before discussing the relationship between blastocyst grade and developmental potential, the technical side of blastocyst grading warrants discussion. In a comprehensive assessment of blastocyst grading (Storr *et al.*, 2017), a group of experienced embryologists could consistently rank blastocysts for transfer (kappa = 0.7), but had poor agreement when assigning grades to the ICM and TE (kappa = 0.35), indicating that grading of the individual components of the blastocyst is subjective and prone to inter-observer variability. Of note, the study did not include grade ‘C’ blastocysts.

## Blastocyst Grades and Viability

The focus of grading systems has been on predicting implantation potential—or which embryo is best. There is little evidence on the relationship between grades of low quality blastocysts and their likelihood of leading to a live birth. While the focus has been on ‘useable’ blastocysts (i.e. suitable for transfer and/or freeze), the definition of a viable or useable blastocyst varies considerably. Dokras proposed that blastocysts with ‘degenerative foci’ were grade 3 and considered unsuitable for transfer (Dokras *et al.*, 1993). A quality blastocyst in the Gardner system, which is the foundation for most of the current embryology society grading schemes (Alpha, 2011a), is predicated on the ability to grade the ICM and TE upon expansion to a full blastocyst (stage 3; Gardner and Schoolcraft, 1999). Any blastocyst stage 1 or 2, or ≥3 with a grade ‘C’ ICM or TE, would be deemed low quality. While grade ‘C’ blastocysts are often transferred in fresh cycles, their disposition for freezing or biopsy varies. The Gardner system has resulted in a plethora of studies using the threshold 3BB as the minimal grade for blastocyst usability (Arce *et al.*, 2014; Rubio *et al.*, 2014; Comstock *et al.*, 2015; Munch *et al.*, 2015). In studies using the newer simplified grading systems, similarly grade 3/C blastocysts are often not frozen or biopsied (Restelli *et al.*, 2014; Stone *et al.*, 2014; Hardarson *et al.*, 2015; Richardson *et al.*, 2015; Fawzy *et al.*, 2017). Since low quality blastocysts can lead to live births (Capalbo *et al.*, 2014; Wirleitner *et al.*, 2016; Irani *et al.*, 2017) with normal obstetric and perinatal outcomes (Bouillon *et al.*, 2017), this bias against freezing grade ‘C’ blastocysts has limited the establishment of a lower threshold for viability.

Many clinics adhere to a strict grading policy that discards grade ‘C’ blastocysts, yet there are several studies that provide evidence of their potential. The most compelling evidence for freezing low quality blastocysts is that single embryo transfer of expanded blastocysts with grade ‘C’ ICM or grade ‘C’ TE resulted in live births at rates that, while lower than top quality blastocysts (34.1 versus 46.8%), resulted in 109 live births that had similar obstetric and perinatal outcomes compared to grade ‘A’ or ‘B’ blastocysts (Bouillon *et al.*, 2017). With the advent of PGS, clinics are biopsying, freezing and transferring blastocysts that contain ‘C’ ICM or TE, are stage 1 or 2, or develop on Day 7 (Capalbo *et al.*, 2014; Minasi *et al.*, 2016; Christodoulou *et al.*, 2017; Irani *et al.*, 2017). Grade C blastocysts are often euploid (Capalbo *et al.*, 2014; Christodoulou *et al.*, 2017; Irani *et al.*, 2017) and result in live births (Wirleitner *et al.*, 2016; Bouillon *et al.*, 2017), although they may also result in more miscarriages (Irani *et al.*, 2017). These reports provide sufficient evidence that viable blastocysts are excluded from use either because of low quality or by ending culture on Day 6 using conventional grading and selection.

## Blastocyst Grades and Rank Order

Numerous studies report on the relationship between blastocyst grade and implantation. All studies are limited by their retrospective nature and the inherent biases of embryo selection at each clinic, not to mention the high degree of inter-user variability when grading the ICM and TE (Storr *et al.*, 2017). Nonetheless, studies claim the degree of expansion (Van Den Abbeel *et al.*, 2013), ICM grade (Subira *et al.*, 2016; Irani *et al.*, 2017) or size (Richter *et al.*, 2001; Almagor *et al.*, 2016) or TE grade (Ahlström *et al.*, 2011, 2013; Honnma *et al.*, 2012; Hill *et al.*, 2013) are predictive of implantation. In contrast, in a study of euploid embryos, none of the blastocyst features were related to implantation potential (Minasi *et al.*, 2016).

**Figure 1:**
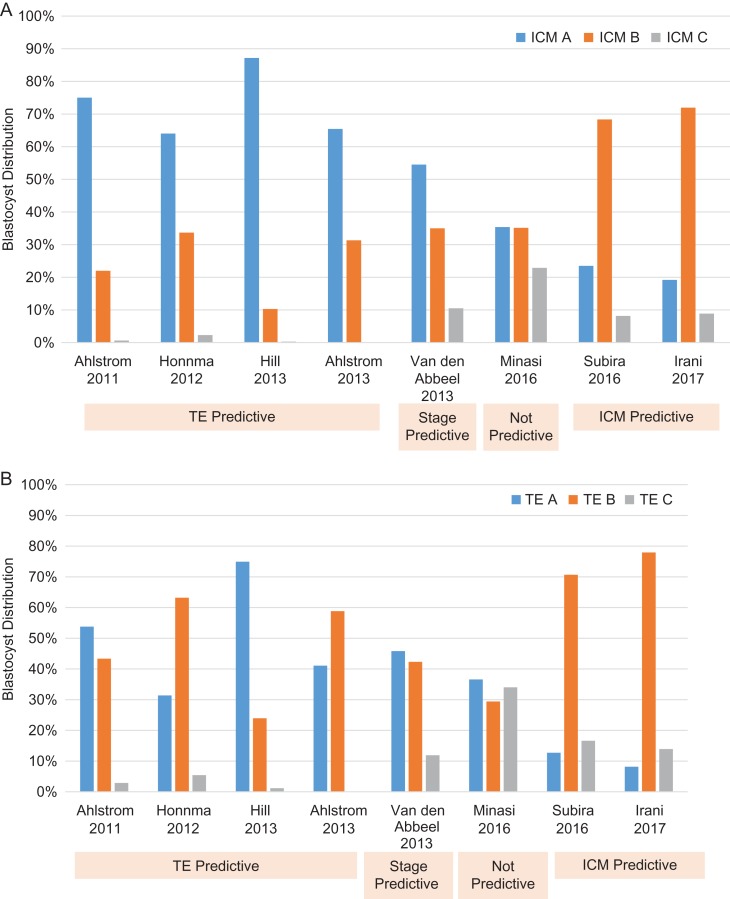
Distribution of grades of blastocysts for studies reporting transfer outcome versus blastocyst grade. Inner cell mass (ICM) (**A**) and trophectoderm (TE) (**B**) grades of blastocysts are shown. Ahlström *et al.* (2011, 2013), Honnma *et al.* (2012) and Hill *et al.* (2013) indicate TE is predictive while Subira *et al.* 2016 and Irani *et al.* 2017 indicate ICM is predictive. Van den Abbeel *et al.* (2013) favors degree of blastocyst expansion while Minasi *et al.* (2016) found none of the features predictive of implantation for euploid blastocysts.

A closer analysis of these retrospective studies illustrates a remarkable difference in grade distribution (Fig. 1). First, grade ‘C’ ICM or TE were mostly absent from the two groups of studies that found ICM or TE predictive, thus making the grading system effectively a two-level system. The fewer the grades, the less discriminatory power is provided. Discriminatory power is weakened further when the grading appears to be biased in favor of one component. The four TE-predictive studies graded the ICM as ‘A’ for 64–87% of the cycles versus the TE as ‘A’ for 22–64% of cycles (Ahlström *et al.*, 2011, 2013; Honnma *et al.*, 2012; Hill *et al.*, 2013). Since the majority of blastocysts had grade ‘A’ ICM, the value of its grade was likely lost. In contrast, while the two ICM-predictive reports (Subira *et al.*, 2016; Irani *et al.*, 2017) had similar high levels of grade ‘B’ ICM and TE embryos (70% average), there were nearly twice as many ICM grade ‘A’ (20%) as TE grade ‘A’ blastocysts (10%). These observations suggest that observed relationships between grades and outcomes were due either to bias in blastocyst grading and selection for transfer, perhaps related to differences in embryologist training or time of grading relative to insemination, or differences in blastocyst quality among clinics. These observations cast considerable doubt on our ability to use morphology to rank blastocyst implantation potential.

Rank order can also be influenced by day of blastocyst formation, and its impact relative to blastocyst grade is unknown. Most early reports on differences in Day 5 versus Day 6 blastocysts were limited by the freezing method used and double embryo transfers (reviewed by Sunkara *et al.*, 2010). Although there was a trend to increased live birth rate with Day 5 blastocysts, this effect was lost when the analysis included blastocyst quality. More recent studies indicate that Day 5 blastocysts may be superior to Day 6 blastocysts, although this difference appears to be lost when euploid embryos are transferred (Capalbo *et al.*, 2014; Piccolomini *et al.*, 2016). The developmental potential of Day 7 blastocysts relative to Day 5/6 blastocysts is likely reduced but the data available are limited (Richter *et al.*, 2006; Hiraoka *et al.*, 2009; Kovalevsky *et al.*, 2013).

Embryologists are left without a clear case for rank order based on embryo quality and day of freezing. The decision for which embryo to warm is easy with embryos of similar quality frozen on different days but lacks any evidence to weight blastocysts of different quality versus day of freezing.

## Where Do We Go From Here?

Clinical strategies such as freeze-all and PGS push biology and the embryology laboratory to their limits. Though the merits of freeze-all are well established (Blockeel *et al.*, 2016), the risks to the embryo, and thus the care of the patient, are mostly undocumented. These risks include the possibility that culture conditions cannot support an embryo’s development to the blastocyst stage, the chance that a viable embryo is graded as poor and not used, and that a low quality blastocyst won’t survive the freezing/thawing process. Since PGS typically includes freeze-all, the same risks apply plus the risk of misdiagnosis owing to mosaicism and the unknown risk of biopsy and freezing on poor quality blastocysts. The waters are truly treacherous for low quality, slow growing blastocysts.

Not all patients present the same—in fact they present on a continuum with scarce to abundant resources in terms of eggs, embryos and finances. Most clinics practice a ‘one-size fits all’ approach to keep the process simple. This approach works well for patients with an abundance of good quality blastocysts by limiting the number of choices. The approach also works well for patients wanting to optimize success rate per transfer or those who wish to minimize the risk of miscarriage. However, for many patients a single ART cycle is their one chance at biological parenthood and if the result is a low quality blastocyst, they should know that we lack certainty when determining its disposition.

Acknowledging our uncertainty about blastocyst viability and developmental potential introduces a complexity into clinical practice that can have undesirable consequences. Unintended consequences of a more liberal blastocyst utilization policy include a decrease in frozen embryo transfer live birth rates, a potential increase in miscarriages, and a significant increase in cost and burden in the ART laboratory and to the patient: costs that may ultimately lead to the patient discontinuing their care. In order to practice individualized medicine in this environment, we need to spend more time counseling patients prior to their cycle to develop a plan that fits the patient’s needs.

The ART laboratory is impacted in many ways by this acknowledgment, and steps should be taken to minimize the effect. The first step is to acknowledge that we do not, and possibly will not, know whether good quality blastocysts (grade 3BB or better) have significantly a different implantation potential. The amount of time and effort spent grading and tracking ICM/TE grades is likely wasteful and can be minimized. To this end, the simplified grading systems that eliminate the A versus B ICM/TE distinction are a good start (Richardson *et al.*, 2015; Wirleitner *et al.*, 2016), as well as efforts to measure blastocyst diameter as a quantification of expansion (Bodri *et al.*, 2015). Refinement of criteria for grade ‘C’ embryos and the relationship to implantation potential should be next.

We crave simplicity and certainty—blastocyst grading is neither. In our pursuit of defining the ‘best’ embryo to transfer, we have neglected to define ‘viability’ of low quality and slow growing blastocysts.

## Recommendations

### Freeze/biopsy Day 7 blastocysts

Available evidence from several reports indicates that the speed of development to the blastocyst stage is variable and that slow growing blastocysts, while likely of lower developmental potential with higher aneuploidy and miscarriage rates (Minasi *et al.*, 2016; Irani *et al.*, 2017), are often euploid and result in live births. Our ability to preserve viability with vitrification and to reset embryo:endometrium synchrony via a frozen embryo replacement cycle are keys to the successful use of Day 7 blastocysts. More studies are needed to adequately assess the developmental potential of Day 7 blastocysts in order to provide patients with realistic expectations and to establish implantation and live birth rates. Inclusion of grade ‘C’ blastocysts adds an additional layer of uncertainty in the context of Day 7 blastocysts and requires careful consideration. Furthermore, since slow development in older patients is associated with higher aneuploidy rates (Campbell *et al.*, 2014; Piccolomini *et al.*, 2016), guidelines for freezing slow growing blastocysts should be relative to age.

### Refine grade ‘C’

In most systems, grade ‘C’ blastocysts have either poor/no ICM or poor/few TE cells, or both, yet we know little about the importance of the ICM versus TE in the context of ‘C’ grades. The available evidence indicates that more objective measures of ICM and TE morphology are needed when quality is low. Artificial intelligence (AI) analysis of blastocysts may provide this evidence (Filho *et al.*, 2012; Rocha *et al.*, 2017). While AI has promise, there is an urgent need to develop more consistent and objective manual methods of assessment. Several candidates exist, including the diameter of the blastocyst, number of TE cells in the largest cross section, and size of the ICM (Richter *et al.*, 2001). With more objective measures, the relative developmental potential for embryos that are often called ‘non-viable’ can be determined and incorporated in the decision to freeze or biopsy.

### Develop patient education materials addressing viability assessment in order to provide an individualized plan, particularly for patients with few embryos

Most patients present to clinics with infertility and most patients older than 38 years have few embryos for selection. The impact of uncertainty—whether of the ability of an embryo to develop into a blastocyst *in vitro*, survive vitrification with the ability to implant, or the results of aneuploidy screening—should be addressed so that patients are aware that the information available for a decision on the viability of an embryo is limited. Counseling should incorporate patient-specific factors that affect blastocyst rate, such as maternal age (Thomas *et al.*, 2010) or number of oocytes (Stone *et al.*, 2014), although at present the impact of patient-specific factors on the number or prognosis of grade ‘C’ blastocysts is unknown.

The number and quality of an embryo cohort vary considerably among patients, variation that when juxtaposed with each patient’s unique journey means a ‘one-size fits all’ plan for low quality blastocysts is unrealistic and is not recommended. For patients with many high quality embryos, low quality blastocysts carry additional emotional and financial costs. Treatment fatigue and dropout occurs and limits fertility treatment success (Gameiro *et al.*, 2012), indicating that repeated transfers of embryos with low implantation potential may have unintended consequences that could be avoided by undergoing another oocyte retrieval to obtain blastocysts with higher developmental potential. For some patients, surplus embryos that are not wanted are an emotional and ethical burden. Thus the decision to freeze low quality blastocysts should be made on a case-by-case basis, irrespective of the quality or size of the embryo cohort.

### Incorporate poor quality blastocysts and day of blastulation into KPIs

Regarding embryo utilization KPIs, the journey has just begun. The new consensus document on laboratory KPIs suggests a minimum overall and good quality blastocyst development rate on Day 5 of 40 and 30%, respectively, and aspirational benchmarks of 60 and 40%, respectively (ESHRE SIG/ALpha, 2017). The introduction of KPIs for blastocyst rates is long overdue and is a good starting point to address the uncertainty in blastocyst developmental potential. For clinics that freeze or biopsy slow and/or low quality blastocysts, inclusion of these blastocysts will yield higher KPIs relative to industry standards. The contribution of low quality blastocysts to a high blastocyst rate may itself be an important performance indicator: the significance of number of low quality blastocysts relative to good quality blastocysts is not known and merits further assessment.

### Report clinic outcome data for live births per retrieval performed

The decision to freeze, biopsy and transfer low quality blastocysts will impact clinical outcomes, resulting in a decrease in live birth rates per transfer but likely an increase in cumulative live birth rates per retrieval. Clinic league tables that only show pregnancy/live birth per transfer will reflect poorly on clinics that incorporate more low quality frozen blastocysts. Publishing live birth rates per retrieval will minimize this impact.

## Conclusions

In the past two decades, the ART laboratory has played a central role in improving IVF outcomes, with the wide-scale adoption of blastocyst culture a key development. However, we still largely assess blastocyst quality on appearance rather than viability. We now know that blastocysts that rate poorly using conventional scoring can result in normal live births, indicating that ART clinics should re-evaluate the threshold criteria used for blastocyst viability. The impact of incorporating blastocysts previously relegated as too poor to transfer on patient care cannot be overstated, both for patients who may expend valuable time and resources on possibly futile care, as well as for those patients for whom the grade ‘C’ blastocyst results in a healthy child. In order to practice evidence-based, personalized medicine, ART clinics need a revised blastocyst grading scheme, practice guidelines and patient education materials that address our limited ability to predict blastocyst viability. The answer to ‘Is this blastocyst good enough to use?’ may depend on the patient’s circumstances, values, and resources, as much as on the embryo itself.
